# Applications of Non-invasive and Novel Methods of Low-Field Nuclear Magnetic Resonance and Magnetic Resonance Imaging in Aquatic Products

**DOI:** 10.3389/fnut.2021.651804

**Published:** 2021-03-19

**Authors:** Xin-Yun Wang, Jing Xie, Xin-Jun Chen

**Affiliations:** ^1^Shanghai Engineering Research Center of Aquatic Product Processing and Preservation, Shanghai, China; ^2^Shanghai Professional Technology Service Platform on Cold Chain Equipment Performance and Energy Saving Evaluation, Shanghai, China; ^3^National Experimental Teaching Demonstration Center for Food Science and Engineering, Shanghai Ocean University, Shanghai, China; ^4^College of Food Science and Technology, Shanghai Ocean University, Shanghai, China; ^5^College of Marine Sciences, Shanghai Ocean University, Shanghai, China

**Keywords:** low field nuclear magnetic resonance, aquatic products, magnetic resonance imaging, quality, non-destructive testing, real-time monitor

## Abstract

Aquatic products, such as fish, are popular throughout the world due to their satisfying flavor characteristics as well as rich animal nutrition, and they provide high-value food therapy, but they are easily oxidized and spoiled. It is necessary to detect aquatic products through rapid and accurate technology. Low-field nuclear magnetic resonance (LF-NMR) and magnetic resonance imaging (MRI) have been widely used in the aquatic product industry due to their sensitivity, fast analysis, non-destructive nature and low cost. The applications of LF-NMR in the measurement of aquatic product quality and nutrients (water, fat, and protein) are summarized in this paper. Applications in aquatic products have been shown to depend on deep processing, storage and authentication. This review discusses the application of MRI technology in the quality control of aquatic products. Therefore, this review will guide the application of the aquatic products industry and aims to supply the reader with both the theory of the method and practical applications of the method for use as a rapid and non-destructive technology in scientific research and the industrial industry.

## Introduction

Aquatic products, a relatively high-nutrition and economically valuable food, have become a popular food among consumers and producers around the world (1, 2). Due to their high water content, the flesh of aquatic products spoils easily. Therefore, to ensure the quality and safety of aquatic foods, non-destructive and fast technologies are applied with quality monitoring since they maintain the quality of the traditional character, including texture, taste, and flavor. At present, it is reported that 30% of aquatic products cannot be consumed each year due to spoilage, which accounts for 25% of total agricultural losses (3). Therefore, it is essential to attend to the quality monitoring of aquatic products during production, storage, processing, and transportation.

With the introduction of advanced technologies, low-field nuclear magnetic resonance (LF-NMR) has become a powerful tool that is gradually being applied for the detection of water content, distribution, and migration in aquatic products (4, 5). It is considered to be an accurate and non-destructive method for visualizing the internal food structure (6, 7). Meanwhile, magnetic resonance imaging (MRI) has been widely used in life sciences and food science research (6, 8). Moreover, the transverse relaxation time (T_2_)-weighted nuclear magnetic resonance (NMR) signal-obtained relaxation time has been proven to be very informative for water dynamics and its correlation with quality changes, as has been demonstrated using the chemometrics model to predict the shelf life of aquatic products (9–12).

In this paper, our review covers the progress and applications of LF-NMR in aquatic products for the determination of water and fat as well as protein. This review has summarized the latest application progress of LF-NMR in deep processing, storage and authentication. The applications of MRI technology in the non-destructive visualization of aquatic products are introduced. Finally, the potential of using LF-NMR/MRI as a technique for non-destructive testing in aquatic products was shown, which will strongly contribute to applications and developments in the aquatic product industry.

## Overview of Low-Field Nuclear Magnetic Resonance Technology

NMR spectroscopy probes into the interaction of a nucleus with an applied external magnetic field (13) and was initially used to illustrate the structure of molecules and their chemical properties in the 1970s (14). It can qualitatively and quantitatively analyze the composition and structure of organic and inorganic materials (15). NMR can be used to analyze the behavior of NMR-active nuclei (i.e., ^1^H and ^13^C, which are most commonly used for food and processed product applications) (16) in a magnetic field or exposed to pulsed radiofrequency (RF) irradiation (17). Relaxation is the complicated process whereby nuclei transition from an excited state [owing to the splitting of the nuclear spin levels (Zeeman effect) of an applied magnetic field] to equilibrium (18).

**Figure 1:**
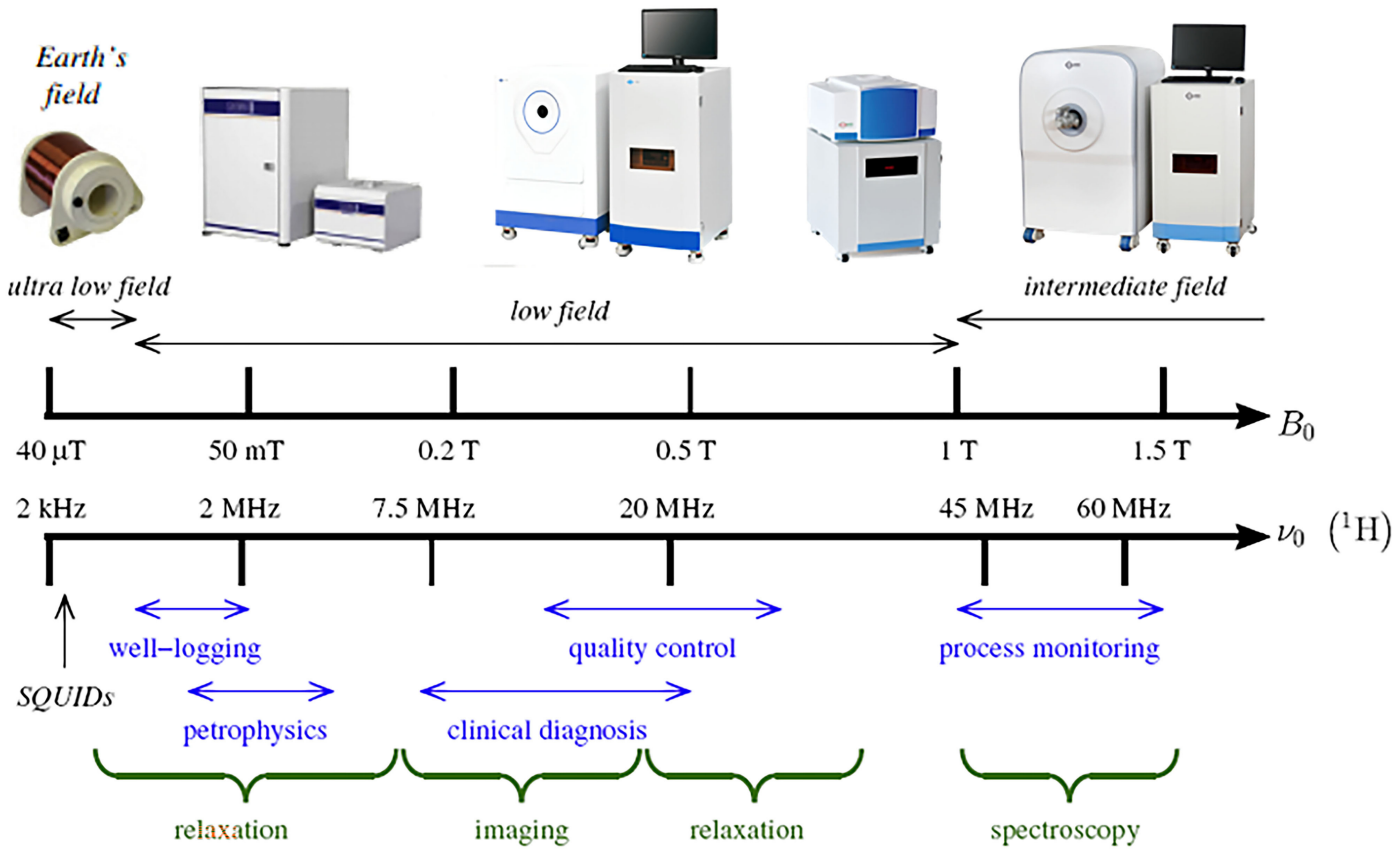
Schematic illustration of the strength of the magnetic field.

**Figure 2:**
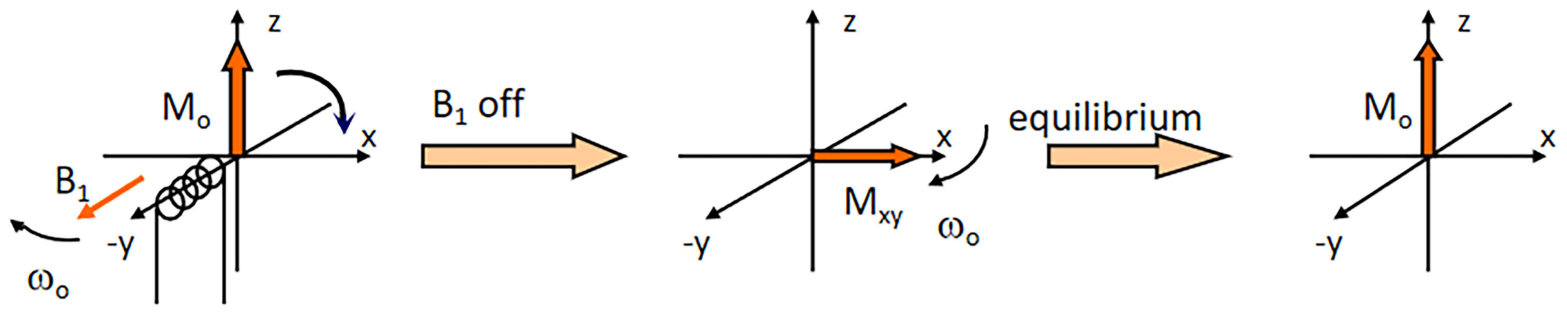
Schematic illustration of the relaxation time principle.

According to the strength of the magnetic field, NMR technology is divided into high-field NMR (≥1.0 Tesla, T: unit indicating the magnitude of a magnet), middle-field NMR (0.5–1 T) and low-field NMR ( ≤ 0.5 T) (Figure 1). High-field NMR technology has the advantages of high sensitivity, high resolution and a high signal-to-noise ratio; however, a significant limitation of the related instruments is that they are expensive and require periodic replenishment of liquid nitrogen (19, 20) (Figure 2). The price of high-field NMR is 10 times that of LF-NMR. Compared with high-field NMR technology, LF-NMR not only requires low instrument costs and no special sites for installation but also contains shields inside the instrument and does not need refrigeration. LF-NMR belongs to the submicroscopic field (between molecules) *via* spin-lattice relaxation (i.e., longitudinal relaxation time, T_1_) and spin–spin relaxation (i.e., transverse relaxation time, T_2_). NMR relaxation measurements of water protons provide a great deal of information about the dynamics of water (21). The RF pulse is assumed to resonate with the hydrogen proton. Some of the low-energy hydrogen proton absorption energy transitions to a high-energy state. After the RF pulse disappears, the hydrogen proton returns in a non-radiative manner to the ground state and reaches a Boltzmann equilibrium; the time required for this process is the relaxation time, and it is used to obtain kinetic information between molecules (22–24).

In 1973, Dr. Paul C. Lauterbur first acquired magnetic resonance images by spatial coding. This established a branch of magnetic resonance imaging (MRI) (25). MRI is a non-destructive and non-invasive detection technology that visually reflects the internal structure of a sample. MRI has significant importance in the real-time monitoring of quality changes in aquatic products during processing, transportation, and storage.

The number of references analyzing foods with LF-NMR and MRI over the last 5 years shows that there has been an increasing number of publications over time, indicating that LF-NMR and MRI have been used more frequently in food research. Furthermore, many publications have proven that LF-NMR and MRI techniques have played a key role in investigating the most abundant chemical components (water, protein, and fat), deep processing, storage, and authentication of aquatic products. The number of scientific works regarding the use of LF-NMR for chemical components increased from 163 papers to 291 during the period 2017–2020. The current paper covered most studies that shed light on the deep processing and storage of aquatic products. The number of published works on deep processing and storage of aquatic products increased from 33 papers to 82 during the period 2017–2020.

## Applications of LF-NMR in Aquatic Product Non-Destructive Testing

Aquatic products are easily oxidized and spoiled. Traditional measurement methods used to analyze aquatic product quality are not only time-consuming ones but also destroy the sample. With the increasing demand for international aquatic products, it is necessary to develop a fast, non-destructive detection technology. According to these technical requirements, LF-NMR was used for the detection of aquatic product quality and opportunities.

### Measurement of Water in Aquatic Products

**Figure 3:**
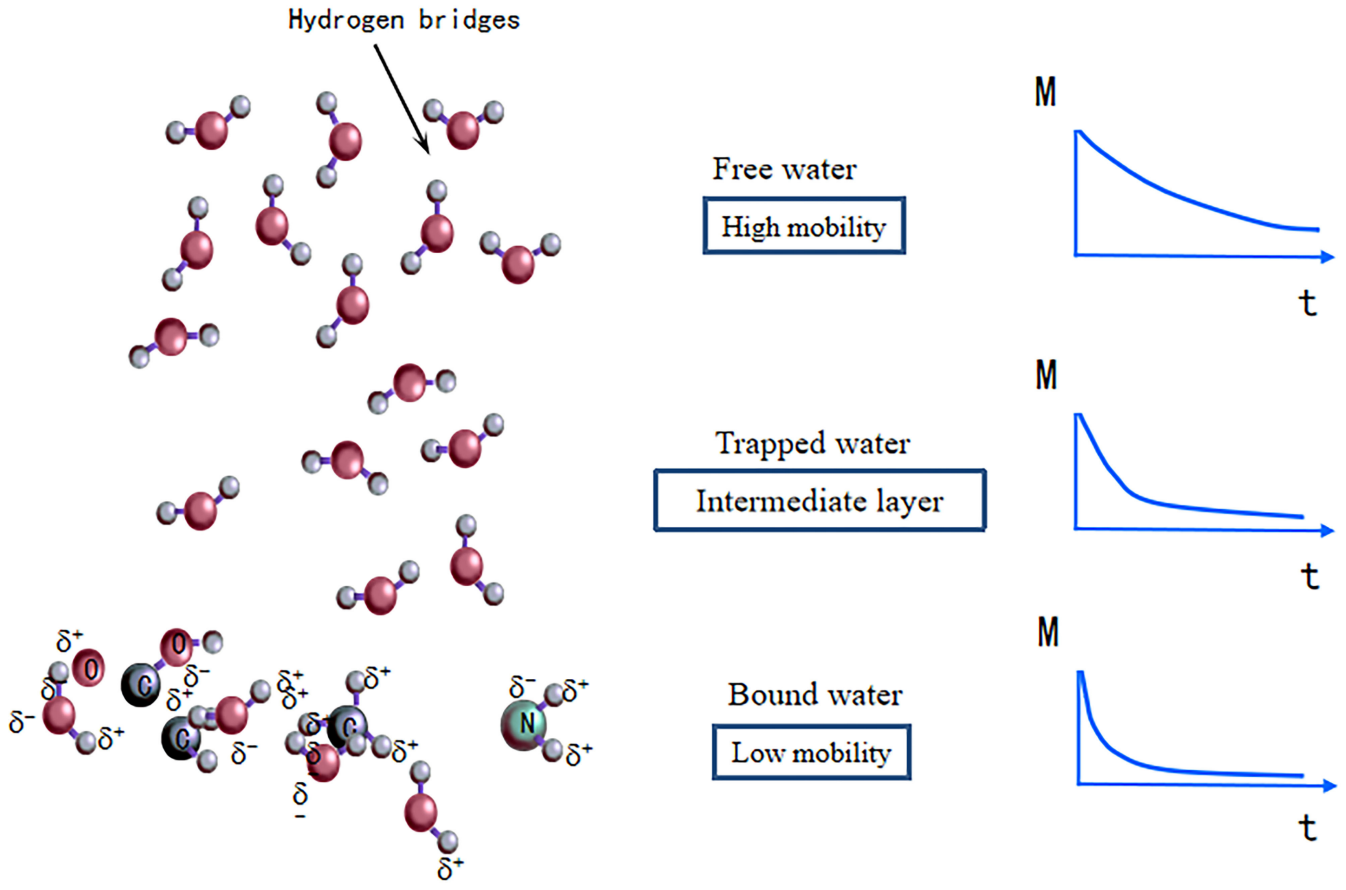
Schematic illustration of the three water phase models (bound water, trapped water, and free water).

Water is the most abundant chemical component in aquatic products, and water content change is one of the important indicators used to evaluate the quality of aquatic products (26). Based on the state of the water, it can be divided into bound water (tightly bound to macromolecules, such as proteins), trapped water (within the myofibrillar structure), and free water (the water outside the myofibrils) (24) (Figure 3). There are a variety of traditional moisture measurement methods, such as the direct drying method, vacuum drying method, distillation method, and Karl Fischer method. Traditional measurement methods are time consuming and can only measure the water content, while the state and distribution of water cannot be determined. LF-NMR can be applied to the detection of moisture content, distribution, mobility, and water-binding state in aquatic products. The T_2_ relaxation time can be detected by LF-NMR, and water migration and the water content are analyzed according to the changes in T_2_ in aquatic products. At present, LF-NMR has been reported in the literature for analyzing water dynamics and its correlations with the water holding capacity, texture, and flavor of fish (10, 27).

The changes in the water state of dried sea cucumber during rehydration, as well as the interaction of water with the surrounding macromolecules, can significantly affect T_1_ during LF-NMR analysis (28). The moisture content was determined by T_2_ transverse time. The proper presoaking and rehydration times were estimated to be 24 and 96 h, respectively. The rehydration ratio of dried sea cucumber was analyzed by principal component analysis (PCA). There was a good correlation between water content and chewiness. The dry and salt-containing sea cucumbers were clearly distinguished on the PCA score map. LF-NMR can indirectly reflect the texture characteristics of aquatic products, and the results were consistent with the texture parameters of aquatic products. The change in the quality of the wet surface of kelp was recorded as the T_2_ relaxation time during storage at room temperature, 4 and −18°C. The amount of trapped water on the wet surface of kelp increased with the extension of storage time. The critical quality control period was between 28 and 35 d at room temperature, and the critical periods of quality control at 4 and −18°C were between 35 and 42 d and between 49 and 56 d (29). The above results indicated that LF-NMR could be used to measure water changes. Water content can be used as a reference standard for predicting the shelf life of aquatic products. Liu et al. (30) used LF-NMR combined with water-holding capacity, Ca^2+^-ATPase activity and texture analyses, and this combined method provided a great correlation with trapped water (T_21_), water-holding capacity, free water (T_22_), and elasticity. The results were consistent with those of Wang et al. (31), who studied the mechanism of water changes by LF-NMR. This result suggested that T_21_ was directly related to the water holding capacity. The elasticity of aquatic products was consistent with the variation trend of free water content, and water changes would affect the flavor of aquatic products.

### Measurement of Fat Content in Aquatic Products

Fat is an essential nutrient, and fat content can thus be used to evaluate the quality of aquatic products. There are a variety of traditional methods used for fat content determination, such as the oil weight method, residual method, Soxhlet extraction method, Babcock method, and Gabb's method (32). Traditional measurement methods can be complicated, inaccurate, time-consuming, and labor-intensive. However, samples are qualitatively analyzed by these traditional measurement methods, so corresponding chromatographic peaks must be compared using known data or combined with mass spectrometry/infrared spectroscopy data. A significant limitation of mass spectrometry/infrared spectroscopy methods is their lowered sensitivity for less polar compounds (e.g., hydrocarbons and organometallic compounds). In the quantitative analysis of a sample, standard substances are required to calibrate the output signal before detection, the cost is high, a professional technician is required, and mass spectrometry/infrared spectroscopy is also time-consuming and laborious. The national standard GB/T 31743-2015 allowed for the direct detection of solid fat content by NMR, and the solid fat signal and liquid fat signal observed for the sample were directly determined by NMR and calculated to obtain the solid fat content (32).

At present, LF-NMR has been applied as a technique to rapidly and non-destructively detect fat content. LF-NMR combined with stoichiometry could be used to analyze the fat content of aquatic products, which could be characterized by T_1_-weighted imaging and the T_2_ relaxation time (33, 34). The fat content of salmon was quantitatively analyzed by pulsed nuclear magnetic resonance relaxation signals (range of 90–182 g kg^−1^), and this technique was combined with the novel software “Norwegian mass cutting (NQC)” to obtain a “fat image.” Therefore, the NMR spectrometer monitored the fat changes online and can thus be expected to be an increasingly popular research tool (35). Moreover, LF-NMR is more convenient and accurate than traditional Soxhlet extraction (28, 36). The relationships between water migration and the fat content of aquatic products were investigated with were investigated. Fat content was visually reflected by changes in T_1_-weighted imaging, and CPMG echo peaks and fat prediction models, including principal component regression (PCR) and partial least squares regression (PLSR), have been rapidly established. LF-NMR, which is combined with chemometric methods, has been used for the quantitative analysis and quality control of fat content in aquatic products.

### Measurement of Protein in Aquatic Products

Protein is the main component of aquatic products. Three kinds of proteins from aquatic products are myofibrillar protein, myogen, and matrix protein. The quality changes of aquatic products are mainly caused by the degradation of the myosin heavy chain, α-actin, actin, and tropomyosin. The combination of physical, chemical, and microbiological reactions resulted in a decrease in the freshness of aquatic products (37–40). There are a variety of traditional methods used for analyzing proteins, such as SDS-polyacrylamide gel electrophoresis, mass spectrometry, and near-infrared spectroscopy (27, 41–43). These techniques are complicated, time-consuming and laborious. Many factors that influence protein changes and are of significant importance are protein oxidization and degradation. At present, the T_2_ transverse relaxation time determined by LF-NMR can reflect the moisture distribution and migration in the intramyofibrillar space and extramyofibrillar water population. Water molecules are capable of interacting with surrounding surface proteins, leading to relaxation decay rate changes and relaxation declines (44). Meanwhile, the changes in myofibrillar proteins were analyzed from the perspective of water migration and combined with traditional methods used to detect proteins, enzyme activities, texture, and other indicators to evaluate the quality of the aquatic product (27).

Based on the indicators of emulsifying activity, WHC and LF-NMR, the differences in the fatty acid compositions and the acylglycerol structures of the lipid phase significantly affected the emulsifying capacity of the myofibrillar proteins (45). The results above indicated that LF-NMR could be used to explore the mechanism of protein changes. Ozel et al. (45) applied the T_2_ transverse relaxation time to investigate the effects of different polysaccharides on the swelling ratios of whey protein hydrogel composites. LF-NMR is capable of monitoring the hydrogel swelling structure. In addition, LF-NMR provided more information on swelling mechanisms than conventional methods, establishing its potential for further investigation. Greiff et al. (46) used LF-NMR to study the effects of different concentrations of salt additives on the protein structure of carp surimi. As the concentration increased, T_21_ and T_22_ gradually increased, and the cooking loss rate and WHC decreased because the interaction between water molecules and proteins in the muscle fibers was suppressed. The LF-NMR results showed that the difference in protein structures was mainly related to the salt additive concentration.

## Applications of LF-NMR in Deep Processing, Storage and Authentication of Aquatic Products

Note that the signal obtained through LF-NMR studies on the water dynamics of aquatic products comes directly from the water signal of the aquatic product samples. Moreover, the T_2_ transverse relaxation time has been used by many researchers to investigate the water content and distribution in traditional and modern processing, including deep processing, storage, and authentication. The water content and distribution were monitored in real time for aquatic products. LF-NMR can accurately measure water content changes and migration and determine the types of aquatic products and their byproducts. Therefore, it has been gradually applied to the aquatic products industry.

### Applications of LF-NMR in the Preservation of Aquatic Products

Gudjónsdóttir et al. (47) used LF-NMR to study the effects of different fresh-keeping methods (without polyphosphate and brine pickling) on the moisture and salinity of Atlantic salmon filets. The study showed that T_21_ increased as the water content of fish filets increased. T_21_ has a significant correlation with the water content, salt content and water holding capacity. Moreover, similar WHC results were related to water located outside the myofibrillar network (extramyofibrillar) (48, 49). Sánchezalonso et al. (50) used LF-NMR to study the effects of different freezing methods (air blast, liquid nitrogen, and walk-in freezer methods) on the quality of squid slices. The T_2_ transverse relaxation time effectively reflects the water distribution and migration of squid slices in freezer storage due to the wide band of T_21_ within the range 120–360 ms. Moreover, the results showing that these T_2_ changes exist may indicate quality changes because it has been recorded that the freezing rate was optimized and that the temperature used for quality parameters was controlled. Idag et al. (51) studied salt uptake in Atlantic salmon filets and showered that salt uptake was affected by antemortem stress and rigor mortis. The T_2_ transverse relaxation time was used to show that salt diffusion and distribution strongly depended on the fat distribution during the curing process. Da Silva et al. (7, 48) and Ghidini et al. (49) studied the quality changes of aquatic products preserved by different methods (pickling and adding sodium polyphosphate, polyphosphates, or sulphites). They found that the internal aquatic product tissue of proteins degraded, denatured, and aggregated during storage, and the value of trapped water and free water decreased, which was attributed to chemical conversion between water and protein protons. It is well-documented that the T_21_ and T_22_ of frozen shrimp significantly increase with increasing additive concentrations compared with those of the CK group. This further demonstrates the importance of LF-NMR, which can significantly influence the quality of frozen shrimp during preservation. Reseachers (47, 52) studied the effects of injecting additives (salt and protein) and different preservation methods (salt content and modified atmosphere packaging) to evaluate the quality of fish filets during the freezing process and revealed that the infusion of various salts into the fish and the protein distribution were more uniform than those in the untreated group. The addition of protein powder enhanced muscle protein electrostatic force, resulting in increased water amplitude (A_2b_) in myofibrils. Comparing the other processing conditions, the modified atmosphere in the packaging caused the T_22_ migration rate of fish to slow as well, indicating that the modified atmosphere in the packaging had an increasingly protective effect on fish meat losses.

### Applications of LF-NMR in the Heating Process of Aquatic Products

Wang et al. (8) used LF-NMR to reveal that clams treated at 80°C in the water state significantly changed. T_1_ and T_2_ relaxation times are highly correlated with water dynamics in clams during the heating process. Bi et al. (53) used LF-NMR and observed that only one water population was present in sea cucumber (*Stichopus japonicus*) preheated at 40°C for 120 min, and the water did not change dramatically during the heating process. For sea cucumber postheated at 80°C, three distinct populations were shown, and the T_2_ relaxation time of the bulk water decreased dramatically, indicating some changes in internal structures and loss in WHC. A good correlation between the T_2_ relaxation time and TPA analysis parameters was shown for sea cucumber after both preheating and postheating. Therefore, LF-NMR is presented as a new means of assessing quality and understanding structural changes of seafood during the heating process.

### Applications of LF-NMR in the Low-Temperature Storage of Aquatic Products

Wang et al. (10) used LF-NMR to monitor the water mobility of bigeye tuna during low-temperature storage and combined it with texture profile, quality indicator, microorganism and T_2_ relaxation time analyses to establish multiple linear regression equations and predict shelf life. The results showed that LF-NMR could be used to dynamically monitor the water migration of fish during cold storage. Shu-Min et al. (54) studied the water change mechanism of vacuum-packed cucumber juice fish balls during cold storage. The results showed that T_23_ showed a decreasing trend, and free water dynamically changed with extended storage time. Sánchez-Alonso et al. (50) analyzed the quality changes in hake stored at −10°C for 6 months. As the storage time increased, T_22_ and T_21_ decreased, the WHC and viscosity apparently decreased, and the shear force increased, reflecting the juice losses of hake. The relaxation time (T_21_, T_22_), amplitude (A_21_, A_22_), and related quality indicators were used to establish a PLS mathematical model to predict the shelf life. Therefore, LF-NMR can be used as an important detection method for evaluating the quality change of aquatic products during low-temperature storage.

### Applications of LF-NMR in the Dry Storage of Aquatic Products

Water dynamics of abalone (*Haliotis discus hannai Ino*) were assessed using LF-NMR and MRI in which dried abalones were rehydrated for 120 h. There was a good correlation between the hardness, chewiness, rehydration ratio, and T_22_ relaxation time of dried abalone (4). Cheng et al. (55, 56) studied shrimp meat (*Penaeus vannamei*) and *Pacific oyster* (*Crassostrea gigas*) as the research objects and analyzed the drying process by performing LF-NMR. T_21_ decreased, indicating that the water mobility and water freedom of the shrimp and oyster decreased gradually during the drying process. This migration of water from the extramyofibrillar space into the intramyofibrillar space indicates that this shrinkage of myofibrils significantly influences water mobility by decreasing the space available to keep the water due to the drying process, which is implied by the relaxation times of the different water populations. The difference in texture and color and its correlation with water dynamics were evaluated by LF-NMR. In conclusion, the effect of the drying method on the quality of aquatic products was analyzed from multiple angles.

### Applications of LF-NMR in the High-Pressure Treatment of Aquatic Products

Shang et al. (57) used LF-NMR to study pressures of 300, 500, and 600 MPa, causing the relaxation time to increase in sea bass skeletal muscle and making the dynamics of bound water remarkable. However, the pressure of 100 MPa had little effect on the dynamics of bound water, and 200 MPa could cause the relaxation time of bound water to reach a minimum and the bound water to become more stable. High pressure could retain water in different states; thus, the gel-forming capacity and water holding capacity of aquatic products would change. Similar results were obtained in both fresh and smoked salmon samples treated at 100 and 150 MPa in which the T_2_ values of the 150 MPa-treated samples were different to those of the samples treated at the other pressure levels used. High pressure might lead to a slight decrease in dryness, hardness, color, and appearance in fish and affect T_2_ relaxation time, in terms of both fish and relaxation time changes, which could be caused by changes in the structure of the fish proteins. We concluded with LF-NMR that high pressure contributes to some changes related to the texture, WHC and T_2_ relaxation time of fish due to protein denaturation (51). Combined with physicochemical indicator changes and organizational structures, it was observed that high pressure could extend the shelf life of aquatic products.

### Applications of LF-NMR in the Ultrasound Treatment of Aquatic Products

Zhang et al. (58) studied the effect of ultrasonic treatment on the rehydration capacity. As the ultrasonic power increased, T_22_ and T_23_ (A_22_ and A_23_) increased, revealing that the ultrasonic treatment of sea cucumber could lead it to absorb more free water during the rehydration process. The LF-NMR results demonstrated that ultrasonic technology was beneficial to water absorption in aquatic products. Additionally, a study using LF-NMR revealed that ultrasound-assisted immersion freezing reduced the mobility and loss of immobilized and free water in common carp (*Cyprinus carpio*) (59). LF-NMR is an effective way to evaluate the deterioration of fish during ultrasound storage.

### Applications of LF-NMR in the Authentication of Aquatic Products

Liu et al. (60) used LF-NMR to identify fish gills, miscellaneous fish gills, red snapper, and copper pot fish gills. T_2_ was combined with the traditional drying method to determine the moisture content, state, and distribution of surimi, providing a new method for the rapid and non-destructive identification of aquatic products. In addition, T_2_ relaxation time measurements are capable of measuring and mapping prawns injected with different hydrocolloids, such as gelatine, carrageenan, agar, Amophophallus konjac, and xanthan gum. In addition, LF-NMR could be used to study the different states of water in the muscle (head, tail, paw, and back), which could be used to authenticate adulterated prawns (61). Geng et al. (28) and Hassoun et al. (62) used LF-NMR combined with PCA/PLSR to identify dried sea cucumber/salt-dried sea cucumber and adulterated shrimp, and the PCA/PLSR score map can clearly distinguish between the different kinds of sea cucumber and adulterated shrimp. The correlations between NMR parameters, rehydration rate and texture characteristics of dried sea cucumber/adulterated shrimp were analyzed by the linear regression mathematical method. Marciani et al. (63) applied ^1^H NMR to perform data processing and distinguish between wild and farmed salmon, as well as determine their origins, with a high accuracy based on their tissue lipid species and profiles. These studies suggested that they contained complementary functions that would improve the authentication of aquatic products according to their geographical areas and kinds, as well as treatment methods.

### MRI Used for Visual Observation in Aquatic Product Storage and Processing

MRI non-destructively provides a great deal of information on internal molecule distribution for use in development research and estimating the quality of aquatic food (52, 64). X-ray diffraction, scanning electron microscopy, transmission electron microscopy, optical microscopy, and atomic force microscopy have been frequently used in the visual analysis of aquatic products (53). However, these methods cause significant damage to the molecules detected. MRI technology can be used to non-destructively see the sample through sliced images of aquatic products, improving the processing conditions and quality by real-time dynamic information (65).

Geng et al. (28) used MRI and LF-NMR to study the water dynamic changes in the presoaking and rehydration process of dried sea cucumber. MRI imaging results showed that the free water content was more than the trapped water content of the dried sea cucumber in the rehydration process. The rehydrated sea cucumber mainly contained free water. The stoichiometric method combined with MRI image information was used to analyze the moisture content of the aquatic product and predict the shelf life. Zhang et al. (58) found that different powerful ultrasonic methods affected the internal water distribution during the rehydration of sea cucumber. The study showed that the higher the ultrasonic power was, the stronger the signal of hydrogen protons in the rehydration of sea cucumber, the greater the moisture content inside the sea cucumber and the stronger the ability of sea cucumber to rehydrate. This was combined with SEM and pseudocolour images for comparative analysis and the more comprehensive monitoring of aquatic product quality changes. MRI was used to analyze the water distribution and content changes of fish during low-temperature storage (8, 10). The water migration of tuna could be observed visually. Physical and chemical indicators were used to analyze the quality changes and monitor tuna dynamically. Bian et al. (66) used MRI greyscale images and pseudocolour maps to find changes in the water-oil balance of dried saury samples, which was consistent with the sensory evaluation results. At the same time, MRI was combined with sensory scores, microbial indicators and physical-chemical indicators, and the saury's shelf life was predicted. Relying on MRI results is one of the best tools for predicting the shelf life of aquatic products. Wu et al. (67) used MRI to analyze the distribution and content of fish fat tissue under different feeding habits and aquaculture conditions. Therefore, MRI can realize the real-time online, non-destructive and non-invasive detection of moisture mobility in aquatic products to better control the quality of aquatic products.

## Conclusion and Future Research

LF-NMR and MRI are useful for a wide range of applications related to food property and food authentication. While there are many methods that can be used to measure food components, LF-NMR/MRI technology can not only measure the moisture, protein and fat content in aquatic products but also detect quality changes in real time and in a rapid, non-destructive and accurate manner. Examples of applications include using LF-NMR/MRI as a substitute for the conventional method to analyze aquatic products faster; it can distinguish between complex aquatic product types; it can detect water content and monitor water migration; and it can evaluate aquatic product quality to reveal the mechanism of aquatic product spoilage. In addition, nuclear magnetic data can be combined with mathematics and stoichiometry to establish relevant quality models, providing new ideas for evaluating the quality of aquatic products. However, applications of LF-NMR are still relatively scarce, and the applications of combining LF-NMR with related testing instruments are even scarcer due to technical and testing limitations. Nevertheless, LF-NMR/MRI techniques are still applied in aquatic product research and are good candidates for assessing quality control during industrial processes. This future direction has rapidly promoted the applications of LF-NMR, combined with other related instruments, in the real-time monitoring of aquatic products, thereby improving the efficiency of applying non-destructive testing to aquatic products and introducing LF-NMR technology into the corresponding standard system to promote the development of the aquatic product industry.

## Author Contributions

X-YW analyzed the data, wrote the manuscript, and performed the experiments. JX and X-JC made suggestions for revisions and guided the experiments. All authors contributed to the article and approved the submitted version.

## Conflict of Interest

The authors declare that the research was conducted in the absence of any commercial or financial relationships that could be construed as a potential conflict of interest.
